# 

**DOI:** 10.1186/2047-2501-3-S1-I1

**Published:** 2015-02-24

**Authors:** Fernando Martin-Sanchez

**Affiliations:** 1 Health and Biomedical Informatics Centre, University of Melbourne, Australia

## Introduction

When we were invited by the Health Informatics Society of Australia (HISA) in March 2012 to participate in the Scientific Program Committee of a new conference on big data, we had mixed feelings. On one hand, it seemed very exciting to develop this theme in a very appropriate time and try to address aspects which are highly relevant for clinical and biomedical sciences. However, at the same time, it seemed a little bit bold to start this new activity when the concept of big data was still poorly defined and did not yet exist too many (reported) experiences in Australia. Fortunately, we had the precedent of a successful Data Governance conference series and we must say that during the time worked on the organisation of the event, things were on our side. Today there is a greater awareness of the importance of the concept of big data science and many centres are developing projects in this area.

Thanks to the agreement with the journal BMC Health Information Science and Systems (BMC HISS) we have the pleasure to present in this special issue the best papers submitted to this conference. The articles collected in this special issue show the diversity of applications and definitions of big data in health and have been organised around biological levels of increasing complexity from molecules to organisms and from patients to social networks. The process leading to the inclusion of the papers in this special issue started during the review of the submissions for the conference, where three reviewers evaluated the submissions and invited seven submisions to be extended into a full article to be included in this special issue. These submitted full papers underwent a new round of reviews before their acceptance for publication in this special issue.

A first set of papers was focused in the relevance and application of big data at what would be defined as the lower biological complexity levels, from molecules to microorganisms. In their paper B. Goudey *et al *focused in the study of single nucleotide variants at the molecular level and the computing requirements to deal with this source of big data. Expanding the analysis from single nucleotides to genomes and their application in public health L. I. Rusu *et al *presented work showing the increasing importance of the use of next generation sequencing in microbiology and how these data can be considered big data. Continuing in the microbiology area, G. Lopez-Campos *et al *focused their work on the relevance of big data in the study of single microorganisms not only based on the amount but also in the variety of data that have to be managed nowadays for their characterisation and study.

The rest of the selected articles are related with patient data at different levels. The paper by T.D. Nguyen *et al *relates big data with medical imaging presenting all the steps required to develop and run a multimodality research imaging informatics repository. Another source of big data considered by B. Gallego *et al *are hospital electronic data collected routinely in hospitals that can be analysed to improve patient safety. Another area covered in this issue is the relevance of big data associated with the concept of self-quantification, in their work M. Almaki *et al *presented some of the challenges that this approach is posing and its relationship with big data concepts. Finally, and from a social perspective G. Zuccon *et al *addressed the feasibility of using big data available through social networks and other web 2.0 tools such as Twitter to detect and report influenza like diseases as well as the validity of these approaches.

All these articles represent only the tip of the iceberg of the use of big data for health applications. We consider that it is worth to remark the diversity of applications of the works collected in this issue and how they show the diversity of areas where the concept of big data in one way or another is having an impact.

Finally we would like to thank all who submitted abstracts for the conference, the scientific committee program, the authors who submitted the extended version of their works, the reviewers, the HISA staff, BMC HISS and all who have made possible this special issue.

## Declaration

The publication costs for the publication of this paper has been funded by the Health and Biomedical Informatics Centre (HaBIc), The University of Melbourne

This article has been published as part of *Health Information Science and Systems *Volume 3 Supplement 1, 2015: Proceedings of the Health Informatics Society of Australia Big Data Conference (HISA 2013). The full contents of the supplement are available online at http://www.hissjournal.com/supplements/3/S1/.

